# Construction, Validation, and Standardization of the Sexual‐DSMapp Application

**DOI:** 10.1176/appi.prcp.20190047

**Published:** 2020-10-16

**Authors:** Cristian Delcea

**Affiliations:** ^1^ Department of Medicine Iuliu Hat¸ieganu University of Medicine and Pharmacy Department of Advanced Studies in Sexology Sexology Institute of Romania Cluj‐Napoca Romania

## Abstract

**Objective:**

The aim of this research was to construct, validate, and standardize a new tool for assessing sexual dysfunction. The Sexual‐DSMapp is an application that can be used on a computer, tablet, or mobile phone.

**Methods:**

Of the participants (N=800), 400 met *DSM‐5* criteria for clinical dysfunction (the clinical group), and 400 had no dysfunction (the healthy control group). Both groups were 50% male and 50% female. Patients in the clinical group had sexual disorders such as premature ejaculation, erectile dysfunction, diminished sexual desire, orgasm issues, arousal issues, and dyspareunia but no mental, personality, medical, and/or substance use disorders. Average age for both groups was 34, and the average education level was 11.5 years. Participants were from various localities in Romania and were of differing racial‐ethnic groups.

**Results:**

Fidelity of the app's nine scales on Cronbach's alpha (0.975), Spearman's rank‐order correlation coefficient (0.986), and the Guttman scale (0.969) reached maximum threshold, was significant on test‐retest reliability, and reached a Cronbach's alpha average of 0.89 for the whole questionnaire. For the test scales, results from 0.76 to 0.98 were obtained, and significant correlation was shown with other questionnaires. The result of the Kaiser‐Meyer‐ Olkin test for adequate participant sampling was 0.939. Analysis of variance indicated significance (p<0.001). Correlation with similar questionnaires showed significant validity.

**Conclusions:**

The results of this research suggest that the Sexual‐DSMapp can discriminate between clinical and nonclinical sexual dysfunction and can be used to test and evaluate sexual dysfunctions.

## HIGHLIGHTS

- The Sexual‐DSMapp is a new tool for assessing sexual dysfunction that can be used on a computer, tablet, or mobile phone.
- The app can discriminate between clinical and nonclinical sexual dysfunction and can test and evaluate sexual dysfunctions.
- A standardized tool to assess and test for sexual dysfunction will aid advanced technology users, such as specialists in the field of sexology, or patients with sexual dysfunctions.

Traditionally, assessment and testing instruments for sexual dysfunction are developed and standardized as pen‐and‐paper tests to assess categories of sexual disorders and multimodal clinical dimensions regarding sexual dysfunction. Development and standardization of instruments for assessing sexual dysfunction include a clinical conceptual framework and a preexisting theoretical model from which test construction begins (1). Such tools must have good validity, fidelity, and significant interitem and interquestionnaire correlations (2). Most of these assessment tools have good predictability in outlining a psychopathological picture for clinicians, and many have been in use since the 1960s.

Modern information technology (IT) offers an important development in the available options for assessing and testing sexual dysfunction. IT innovations are being increasingly used in several areas of psychological assessment. For instance, virtual reality has proven useful in assessing affective and emotional disorders (3). In addition, mobile apps for assessing and testing psychomotor and neurocognitive disorders in individuals with motor and intellectual disabilities, software applications for individuals with physical and psychological posttraumatic conditions, and software applications used in surgery (4) have been developed. Software applications have also proven effective in sexual education as well as in long‐distance interactions between sexual partners, supported by the Internet, artificial intelligence, and advanced mobile technology.

No software applications are presently available to assess sexual dysfunction. The only software applications currently available in this area are those used to assess individuals with pedophilia in criminal investigations and in forensic medicine departments (5) as well as applications used by urology clinics to assess blood flow to the penis in men with erectile dysfunction (6). Taking into account an estimated worldwide prevalence of 30%–50% for ejaculation disorders (13%–21% among men ages 40 to 80), 4% prevalence of orgasm disorders among men, 5% prevalence of dyspareunia among men (7), 42% prevalence of orgasm disorders among women, 26%–43% prevalence of arousal disorders among women, and 22% prevalence of sexual desire disorders among women (8), the development of a software application in this area is needed to facilitate self‐assessment of sexual dysfunction, thus optimizing sexual satisfaction, and to prevent and treat sexual dysfunction among the general public.

Proceeding from the model proposed by the American Psychiatric Association for the DSM mobile app and taking into account the fact that smartphone users tend to look at their phones approximately 150 times a day (9), I decided to build a smartphone app that would allow users to assess their sexual health, with the goals of preventing and treating dysfunction and optimizing sexual function. The aim of this article was to describe the procedures used to construct, validate, and standardize the Sexual‐DSMapp (S‐DSMapp).

The S‐DSMapp is an interactive computer system that can be used as a tool to provide a qualitative, categorical, indextype evaluation of female and male sexual disorders. The S‐DSMapp is divided into nine scales (female orgasm, male orgasm, excitation, delayed ejaculation, male dyspareunia, female dyspareunia, premature ejaculation, desire in women, and desire in men) for presumptive diagnosis of sexual disorders in accordance with the requirements of the *DSM‐IV‐*TR, the *DSM‐5,* and the *ICD 11.* The app's features facilitate access to the complete *DSM‐IV* and *DSM‐5* diagnostic criteria and establish on the phone or computer the categorical diagnosis of a sexual disorder or the absence of a disorder for clinical and administrative use. Screening via DSMapp meets all the security requirements of IT functionality, with robust discrimination between clinical and nonclinical symptoms, and the application is widely accessible. The app is easy to use; its interface language is adapted to the level of the user's education, enabling users to achieve remission from sexual dysfunction and to optimize their sex lives. The app contains eight items to assess each disorder, and the severity level for each indexed disorder can be calculated. Each question pertains to the criteria for the *DSM* disorders.

## METHODS

### Participants

Participants in this study were 800 individuals who had received specialized health care at the Sexology Institute of Romania between 2016 and 2019. To facilitate creation and validation of the instrument, the participants were classified according to age, sex, and education level.

The sample had completed an average 11.5 years of schooling. The education level varied from grades 10–12 to bachelor's, postgraduate, and doctoral studies. Participants' age ranged from 18 to 78 years, and the average age of the sample was 34.24. There were equal numbers of men (N=400) and women (N=400). The sample consisted of two groups, a clinical group (N=400) representing nine male and female sexual disorders and a nonclinical (healthy control) group (N=400) composed of individuals with no sexual disorders. The number of men and women was the same in each group (N=200).

A majority of participants were from Romania (N=645, 80%), followed by Hungary (N=55, 7%) and Germany (N=23, 3%). Less than 2% of the final sample had originated from other countries.

### Procedure

The Sexology Institute selected 1,340 volunteer respondents who received specialized health care between 2016 and 2019. Participants in the clinical group had sexual disorders but no other mental, personality, medical, or substance‐related disorders. Participants in the nonclinical (healthy control) sample had no sexual disorders and no mental, personality, medical, or substance‐related conditions. Participation was voluntary, and written informed consent was obtained by using a signed research agreement. Approval was obtained from the institutional review board. The participants were informed that they had the right to withdraw from the study at any time. Participation was not motivated by any material or other associated benefits. Participants were selected for either the clinical group or the control group only through the clinical interview.

Of the 1,340 potential respondents, 800 were retained (400 men and 400 women) who met the participation criteria (sex, age, education, clinical or nonclinical status, and country of origin). Some of the 540 excluded individuals had anxiety disorders in addition to sexual dysfunction, and others presented only mental disorders (e.g. anxiety and/or depression) and therefore did not meet the selection criteria, along with individuals who had personality disorders. Other excluded individuals had medical conditions (e.g., chronic prostatitis, diabetes, or cardiovascular or endocrinological issues). The final sample (N=800) was divided into two groups (clinical, [N=400] and nonclinical [N=400]; each group was 50% female and 50% male). The participants were asked to access and respond to the S‐DSMapp in Google Forms.

### Materials and Instruments

The standardized nine‐scale S‐DSMapp questionnaire was input into the Google Forms platform. The S‐DSMapp questionnaire is an interactive informatics system, mediated by a software application, that has been specialized for the qualitative, categorical, index‐type assessment of female and male sexual disorders. The S‐DSMapp is divided into nine scales (female orgasm, male orgasm, excitative, erectile dysfunction, male dyspareunia, female dyspareunia, premature ejaculation, female desire, and male desire). The scales are meant to give a presumptive diagnosis of sexual disorder in conformity with the requirements of the *DSM‐IV‐TR* and *DSM‐5* (10), as well as the *ICD‐10* (11). The app facilitates access to the complete diagnostic criteria of the *DSM‐IV* and *DSM‐5,* allowing for establishment of a categorical diagnosis of sexual disorder on one's phone, tablet, or computer.

The S‐DSMapp meets all security and IT functionality criteria, has good clinical and nonclinical discriminatory robustness, is widely accessible, is easy to use, and has an interface language that can be adapted to the user's level of intelligence and education so that participants can work toward remission of their sexual dysfunction and optimize their sex lives (12). Statistical analyses were performed by using the online platform of SPSS Statistics, version 25.0.

## RESULTS

Two groups were included in the present study: a nonclinical group consisting of healthy control participants (200 men and 200 women) with no sexual, mental, personality, medical, or substance‐related conditions; and a clinical group (200 men and 200 women) with sexual dysfunctions but no mental, personality, medical, or substance‐related conditions (mean±SD variance of overall app score was 2.50±0.040, with a skewness of p<0.001 and a standard error of 0.086). Characteristics of participants are provided in Table 1.

**TABLE 1:** Characteristics of participants (N=800) in study of the Sexual‐DSMapp, by group

Group	Education (years)		Age (years)	
	M	SD	M	SD
Clinical (N=400)	11.50	2.15	34.24	11.12
Nonclinical (N=400)	11.89	2.21	34.26	11.12

### Internal Validity of the S‐DSMapp

*Fidelity.* The fidelity of the nine scales and the entire S‐DSMapp was examined by calculating the Cronbach's alpha internal consistency coefficient for the items of each scale and for the entire questionnaire, which indicated a significant result regarding item correlation in the app. Table 2 shows a high level of internal consistency for the entire app, with a Cronbach's alpha coefficient of 0.975. High levels of internal consistency were obtained by using the Guttman and Spearman‐Brown coefficients. Cronbach's alpha for the two parts of the questionnaire was also high.

**TABLE 2:** Fidelity coefficients of the Sexual‐DSMapp (S‐DSMapp) and similar scales

Scale or questionnaire	Cronbach's alpha	Test‐retest correlation
S‐DSMapp female orgasm	.98	.98
Causal Attribution for Coital Orgasm Scale	.65	.78
S‐DSMapp male orgasm	.96	.96
Orgasm Rating Scale	.88	.92
S‐DSMapp excitative	.96	.97
Sexual Excitation/Sexual Inhibition	.80	.82
Inventory for Women		
S‐DSMapp erectile dysfunction	.71	.79
Sexual Dysfunction Scale	.71	.73
S‐DSMapp male dyspareunia	.80	.83
Sexual Anxiety Scale	.87	.95
S‐DSMapp female dyspareunia	.85	.84
Vaginal Penetration Cognition Questionnaire	.70	.83
S‐DSMapp premature ejaculation	.85	.88
Index of Sexual Satisfaction	.92	.94
S‐DSMapp female desire	.82	.81
Sexual Desire Inventory	.86	.76
S‐DSMapp male desire	.89	.90
Sexual Excitation/Sexual Inhibition	.73	.76
Inventory for Women and Men		

The interscale correlation scores indicated high levels of fidelity (Table 3). The findings also indicated that some groups of variables were highly intercorrelated. For instance, for the male desire, male orgasm, female orgasm, and female desire scales, there were substantial intercorrelations, suggesting the items had strong internal validity.

**TABLE 3:** Interscale correlation scores of the Sexual‐DSMapp scales

App scale	Female	Male		Delayed	Male	Female	Premature	Female	Male
	orgasm	orgasm	Excitative	ejaculation	dyspareunia	dyspareunia	Ejaculation	desire	desire
Female orgasm	1.00	.978	.980	.711	.854	.908	.900	.875	.903
Male orgasm	.978	1.00	.965	.689	.854	.905	.895	.860	.883
Excitative	.980	.965	1.00	.692	.840	.898	.895	.862	.885
Delayed ejaculation	.711	.689	.692	1.00	.540	.608	.605	.585	.822
Male dyspareunia	.854	.854	.840	.540	1.00	.784	.721	.849	.715
Female dyspareunia	.908	.905	.898	.608	.784	1.00	.867	.749	.783
Premature ejaculation	.900	.895	.895	.605	.721	.867	1.00	.746	.780
Female desire	.875	.860	.862	.585	.849	.749	.746	1.00	.760
Male desire	.903	.883	.885	.822	.715	.783	.780	.760	1.000

Cronbach's alpha and the test‐retest coefficients for the app scales were also assessed compared with other similar scales assessing male and female sexual dysfunction. The scale and questionnaire coefficients were significant and above 0.60 in all cases. A Cronbach's alpha coefficient of 0.975 for the entire app was obtained, with a test‐retest correlation of 0.890.

#### Factorial structure

The adequacy of sampling for the total model and for each variable contained within the model were examined with the Kaiser‐Meyer‐Olkin test. Coefficients of 0.80 and above indicated that the sampling was adequate. Furthermore, the significant result of the Bartlett's test suggested homogeneity of variance across groups, indicating the appropriateness of the data for the analysis of variance (ANOVA). A decrease in the factor's eigenvalues occurred after the first factor loading, and the line was practically flat after the second factor was added, indicating high saturation.

#### ANOVA for correlated scores and repeated measurements

In a multivariate ANOVA, the nine scales of the app were compared across the clinical and nonclinical groups. The clinical and nonclinical samples differed significantly across scales, with the significant coefficients suggesting high correspondence among the scales of the app. The equivalence of the Hotelling's trace and Roy's largest root statistics was consistent with the high intercorrelations among the scales. The significant findings of Mauchly's test of assumed sphericity indicated that the assumption of sphericity was violated and that corrections were necessary.

Significant results for tests of within‐subject contrasts were obtained. For instance, in linear factor 1, we obtained F=356, df=2, p<0.001; cubic F=30.52, p<0.001; order 4, F=3.09, df=2, p<0.001; order 5, F=7.74, df=2, p<0.001; order 6, F=14.34, df=2, p<0.001; order 7, F=17.04, df=2, p<0.001; and order 8, (F=25.4, df=2, p<0.001).

Findings from the tests of within‐subjects effects indicated p<.001, demonstrating a substantial effect even when sphericity was assumed. For factor 1, when sphericity was assumed, all three epsilon statistics were significant (F=33.54, df=2, p<0.001), indicating that the between‐subjects effects were significant (F=24.74, df=2, p<0.001).

### Discrimination Between Clinical and Nonclinical Conditions

Table 4 presents the results of an ANOVA comparing the two groups on the total app score. The difference between groups was significant (F=790.01, df=2, p<.001) and indicated that the two groups differed on their average scores on the app. The mean variance of overall app score for both the clinical group and the healthy control group was 2.50±0.40. Table 4 illustrates the significant difference between the two groups with regard to discrimination between clinical and non‐clinical symptoms.

**TABLE 4:** Analysis of variance of clinical and nonclinical groups' total Sexual‐DSMapp scores^a^

Comparison	Sum of squares	df	Mean square	F^b^	p
Between groups	798.989	2	199.747	790.00	<.001
Within groups	201.011	795	.253		
Total	1,000.000	799			

a ^a^The sample consisted of a clinical group (N=400) representing nine sexual disorders (i.e., female orgasm, male orgasm, excitation, delayed ejaculation, male dyspareunia, female dyspareunia, premature ejaculation, desire in women, and desire in men) and a nonclinical (healthy control) group (N=400) composed of individuals with no sexual disorders.

b ^b^df=2.

## DISCUSSION

The results of this research have provided internal validity for the Sexual‐DSMapp. Cronbach's alpha scores for the nine scales were high, indicating substantial internal consistency. High internal consistency was also confirmed by high values for the Spearman‐Brown and Guttman coefficients. The between‐evaluator results obtained by applying the kappa statistic indicated significant correspondence for the items of the app. Internal consistency was shown with significant scores on both between‐item and within‐item correlations, leading to the conclusion that the app has good fidelity with the items it includes. The app was also compared with other similar questionnaires for each scale (13). Substantial correlation was found with other questionnaires, such as the Causal Attribution for Coital Orgasm Scale, Orgasm Rating Scale, Sexual Excitation/Sexual Inhibition Inventory for Women, Sexual Dysfunction Scale, Sexual Anxiety Scale, Vaginal Penetration Cognition Questionnaire, Index of Sexual Satisfaction, Sexual Desire Inventory, and the Sexual Excitation/Sexual Inhibition Inventory for Women and Men. The substantial correlations between the nine scales of the app and other similar questionnaires indicated a good theoretical‐experimental foundation, from which the construction, validation, and standardization of the test began. Significant results were also obtained for test‐retest reliability and resulted in a correlation of 0.89 for the entire app, ranging from 0.76 to 0.98 for the individual test scales.

The app obtained significant scores in its factorial structure regarding interconnected groups of variables (correlation matrix), and the Kaiser‐Meyer‐Olkin test indicated appropriate participant sampling, partial correlations between global variables, and intercorrelations without multicollinearity. Also, in the factorial structure, the first factor of the measured test indicated the robustness of the criterion proposed for assessing sexual dysfunction.

In the ANOVA, significant differences were obtained between the two participant groups on the nine scales of the app. For instance, significant results were obtained by using Mauchly's test of assumed sphericity (14), meaning that the variations of the differences in conditions among the scales (i.e., the levels of the independent variables) were equal. By using the between‐scale contrast method for the app, significant results were obtained for factor 1, indicating assumed sphericity with the Greenhouse‐Geisser and Huynh‐Feldt corrections.

The app also showed robust discrimination between the clinical and control groups with respect to the criterion values in psychological assessment and testing (15). For instance, in the clinical group, results were obtained that indicated discrimination between clinical sexual dysfunctions (premature ejaculation, erectile dysfunction, orgasm disorders in men and women, dyspareunia among men and women, male and female sexual desire, and female arousal) and no clinical dysfunction (in the healthy control group). The results confirmed that the app has good fidelity and validity and may be used to assess sexual dysfunction. The app met all statistical requirements regarding correlations (for the whole questionnaire and for each scale, between items and within each subscale) as well as discrimination between the clinical and control groups.

This research had some limitations. There were no equal samples for the dysfunctions in the clinical group. For instance, among women (N=200), the distribution was not equal for the four sexual disorders (orgasm, arousal, desire, and dyspareunia). A larger number of participants had orgasm and arousal disorders, a small number of participants had desire disorders, and veryfewhad dyspareunia. The same situation appeared with the men in the clinical group (N=200), where more men met the criteria for erectile dysfunction and ejaculation disorder, fewer had desire disorders, and very few had dyspareunia. Test items were limited to the categorical clinical concept in *DSM‐IV* and *DSM‐5,* without outlining a dimension of the disorder, and the small number of items for each scale might index a disorder superficially.

The launch of Unbound Medicine and the American Psychiatric Association's version of the *DSM‐5* diagnostic‐ support app for mobile devices and the Apple Watch is a first step in digitally accessing sexual disorder assessment, but these tools are available only to specialists in the field. However, through the present research and future studies, new tools can be developed for categorial and dimensional assessment of clinical sexual dysfunction that may be used by patients as well.

## CONCLUSIONS

The purpose of this research was to construct, validate, and standardize a new app to assess sexual dysfunction. This app can be used on a computer, tablet, or mobile phone. The results indicated high validity and good fidelity in terms of internal consistency and test‐retest reliability. The large number of participants, as well as the descriptive statistical data obtained, confirm the validity of the test.

Current approaches (16) have claimed that efficiency and willingness to take part in online or smartphone testing of software designed to assess mental and/or sexual disorders is better than those for a pen‐and‐paper version or approaches involving face‐to‐face interaction with an interviewer. Moreover, the digitalized generation needs a new way to assess sexual dysfunction (17). Artificial intelligence is helpful for generating behavior patterns in a test; for providing results to the respondents; and for supervising, guiding, and counseling participants. A standardized tool to assess and test for sexual dysfunction will aid advanced technology users, such as specialists in the field of sexology, or patients with sexual dysfunctions (18, 19, 20).
